# Dynamics of leukocyte telomere length in pregnant women living with HIV, and HIV-negative pregnant women: A longitudinal observational study

**DOI:** 10.1371/journal.pone.0212273

**Published:** 2019-03-06

**Authors:** Sara Saberi, Steve E. Kalloger, Mayanne M. T. Zhu, Beheroze Sattha, Evelyn J. Maan, Julianne van Schalkwyk, Deborah M. Money, Hélène C. F. Côté, the CIHR Team in Cellular Aging and HIV Comorbidities in Women and Children (CARMA)

**Affiliations:** 1 Department of Pathology and Laboratory Medicine, University of British Columbia, Vancouver, British Columbia, Canada; 2 Department of Obstetrics & Gynaecology, University of British Columbia, Vancouver, British Columbia, Canada; 3 British Columbia Women’s Hospital and Health Centre, Vancouver, British Columbia, Canada; 4 Women’s Health Research Institute, Vancouver, British Columbia, Canada; 5 Centre for Blood Research, Vancouver, British Columbia, Canada; University of Newcastle, UNITED KINGDOM

## Abstract

**Background:**

HIV-mediated inflammation and immune activation can accelerate telomere attrition. In addition, antiretrovirals can inhibit telomerase, possibly shortening telomeres. We examined the longitudinal dynamics of leukocyte telomere length (LTL) during pregnancy in a unique cohort of women living with HIV (WLWH) treated with combination antiretroviral therapy (cART), and HIV-negative control women.

**Methods:**

Blood was collected at three visits during pregnancy, at 13–23, >23–30, and >30–40 weeks of gestation, and for WLWH only, at 6 weeks post-partum. LTL was measured by qPCR and both cross-sectional and longitudinal (MANOVA) models were used to examine possible predictors of LTL among participants who attended all three visits during pregnancy.

**Results:**

Among WLWH (n = 64) and HIV-negative women (n = 41), within participant LTL were correlated throughout pregnancy (p<0.001). LTL was shorter among WLWH at first visit, but this difference waned by the second visit. WLWH who discontinued cART post-partum experienced a decrease in LTL. Longitudinally, LTL was similar in both groups and increased as gestation progressed, a change that was more pronounced among women under 35 years. Among WLWH, both smoking throughout pregnancy (p = 0.04) and receiving a ritonavir-boosted protease inhibitor-based regimen (p = 0.03) were independently associated with shorter LTL.

**Conclusions:**

LTL increases as pregnancy progresses; the reasons for this are unknown but may relate to changes in blood volume, hormones, and/or cell subset distribution. While our observations need confirmation in an independent cohort, our data suggest that although some cART regimens may influence LTL, being on cART appears overall protective and that stopping cART post-partum may negatively impact LTL. The effect of smoking on LTL is clearly negative, stressing the importance of smoking cessation.

## Introduction

Women represent more than half of all people living with HIV (PLWH) worldwide [1], and approximately 90% of infections in infants and young children occur through mother to child transmission during pregnancy, labour, delivery or breastfeeding [2]. It is well established that combination antiretroviral therapy (cART) in pregnancy can greatly reduce this risk [3]. Treatment guidelines recommend to initiate cART and continue treatment throughout pregnancy and thereafter in all pregnant and breastfeeding women living with HIV (WLWH), regardless of CD4+ cell count [4–6]. Despite the great success of cART, PLWH remain at increased risk of premature aging and age-related diseases [7–8]. Long term data on the safety of cART exposure during pregnancy remain scarce, but unlike in the past when treatment was often initiated in the second trimester, current guidelines mean that WLWH are likely to be receiving cART at conception and throughout all trimesters of pregnancy.

Cellular aging is associated with many factors, including telomere attrition, mitochondrial dysfunction, and DNA damage [9]. In most cells, telomeres shorten with each cell division, as part of the aging process. Inflammation and oxidative stress can also lead to telomere attrition [10] and these are increased in HIV [11–12]. Telomere length is therefore a marker of the combined effect of these biological processes [13–14]. Telomerase is the enzyme complex responsible for the replication of telomeric DNA during cell division. It is expressed in germ cells, embryonic and adult tissue stem cells [15], placenta [16], hematopoietic stem cells [17] and activated lymphocytes [18], where its activity prevents telomere shortening [19]. In most somatic tissues, telomerase is not expressed and telomeres progressively shorten. When telomeres reach a critically short length, cell senescence or death is induced. Shorter leukocyte telomere length (LTL) has been associated with mortality [20] and increased risk of cardiovascular disease [21]. Some studies have suggested possible links between telomere length/telomerase and reproductive health, including fertility and preterm delivery [22, 23].

Among PLWH, telomere attrition could be modulated by chronic immune activation, inflammation, and oxidative stress [11, 12]. In addition, some HIV proteins have been reported to down-modulate telomerase activity [24, 25]. Telomerase reverse transcriptase (RT) is homologous to HIV RT and may be an unintended target of HIV therapies [26, 27]. The vast majority of cART regimens include nucleoside RT inhibitors (NRTIs) shown to also inhibit telomerase activity both *in vitro* and in cultured human cell models [28–32]. Several NRTIs also shorten telomeres in animal models [33]. In addition, several antiretroviral agents can induce oxidative stress [34–36], which could also negatively affect LTL. Consequently, both HIV infection and its treatment with cART could potentially contribute to accelerated telomere shortening in PLWH.

Although several studies have reported shorter LTL in PLWH, a clear association with cART exposure in humans has not been demonstrated. Indeed, several studies suggest that cART may exert an overall beneficial effect on blood cell telomeres [37–40]. Furthermore, although it is well established that women have longer LTL than men [41–44], nothing is known about how LTL may be affected during pregnancy. Herein, we examined LTL over the course of pregnancy in women living with HIV (WLWH) and HIV-negative women, and explored predictors of LTL, including the effect of cART. We hypothesized that pregnant WLWH would have shorter LTL than HIV-negative pregnant women and that cART would modulate LTL.

## Methods

### Study participants

This was a prospective longitudinal observational study of pregnant WLWH and HIV-negative pregnant women enrolled in two consecutive cohorts, namely the Pregnancy cohort (between 2004 and 2009) and the Children and Women: Antiretrovirals and Markers of Aging (CARMA) cohort (between 2009 and 2012), in Vancouver, Canada. These cohorts, funded through two sequential CIHR grants, had identical inclusion/exclusion criteria and highly similar protocols for data collection and biospecimen processing, and were led by the same investigators. Pregnant WLWH were recruited exclusively from the Oak Tree Clinic at B.C. Women’s Hospital. HIV-negative participants were recruited at BC Women’s Hospital, and through a variety of means, including word of mouth and advertisements posted in strategic areas. Special attention was given to enrolling controls with sociodemographic characteristics similar to those of pregnant WLWH. Inclusion criteria for both WLWH and HIV-negative women were being pregnant at any age with a known HIV status. Of note, the original inclusion criteria for all women was being 19 years of age or older. However, we amended our protocol and IRB to include younger pregnant participants. For WLWH, to be treated with or be willing to take ARVs during pregnancy was the other inclusion criteria. Exclusion criteria included the inability to provide informed consent (language barriers) or to participate in the study (health or social crisis). Enrollment in the study usually took place in the first trimester and participants provided biological specimens at three visits during pregnancy. The first visit (A) took place between 13 and 23 weeks of gestation. The second (B) and third (C) visits were at >23–30, and >30–40 weeks of gestation, respectively. In the case of WLWH, additional visits (with specimen collection) took place at the time of delivery (visit Del) and post-partum, usually between 6 and 8 weeks (visit P-P). Study participants received a $20 honorarium at each visit. All specimens were stored at -80°C until DNA extraction. In cases of repeat pregnancies, only the first pregnancy was included in this analysis. Demographic, clinical, and substance use data were collected at each visit. Details about these variables are described in the S1 supporting information. The table in S1 Table describes the number of available blood specimens at each study visit.

### Ethical considerations

Ethical approval for this study was obtained from the Research Ethics Boards of the University of British Columbia and from the Hospital Research Review Committee of the Children’s and Women’s Health Centre of British Columbia (H03-70356, H04-70540, and H07-03136). All women provided written informed consent. Two of the study authors (JVS and DMM) were also treating physicians for study participants.

### Relative LTL measurement

Relative LTL were measured by a monochromatic multiplex qPCR assay modified from [45] and described in detail previously [46], as well as in the S1 supporting information.

### Statistical analyses

Only study participants who attended all three planned study visits during pregnancy were included in the analyses. Comparisons between groups used Fisher's exact test for categorical variables, and Student t-test or Mann-Whitney test for continuous variables, using XLSTAT version 2012.6.08 (Addinsoft, NY, USA). For the longitudinal analyses of the three pregnancy visits (A, B, and C), multivariate analysis of variance (MANOVA) models were performed to explore the relationship(s) among LTL and the following explanatory variables: maternal age, self-reported ethnicity, income, HIV status, history of HCV infection, substance use throughout pregnancy, weeks of gestation at visit, preterm delivery (gestational age (GA) <37 weeks), as well as cohort (CARMA *vs*. Pregnancy). The same set of analyses was performed on the entire study sample, as well as within WLWH and HIV-negative women separately. Among WLWH, in addition to the variables listed above, HIV specific variables including CD4+ cell count at visit, HIV plasma viral load (pVL) at visit (detectable *vs*. undetectable <50 copies/mL), cART status at visit (on *vs*. off) at visit, and type of cART regimen received during pregnancy (ritonavir-boosted protease inhibitor [PI/r] *vs*. other regimens). Complete definition of variables and procedure for data imputation in case of missing data (e.g. CD4+ cell count and HIV pVL at visit) are described in the S1 supporting information. Variables univariately associated with LTL with p≤0.1 were included in multivariable MANOVA models with LTL as the dependent variable. Independent categorical variables with levels populated with less than 5 observations, were omitted from the multivariable analyses, All MANOVA models were performed using JMP software, v. 12.2.0 (SAS Institute, NC, USA).

## Results

### Study participants

**Fig 1:**
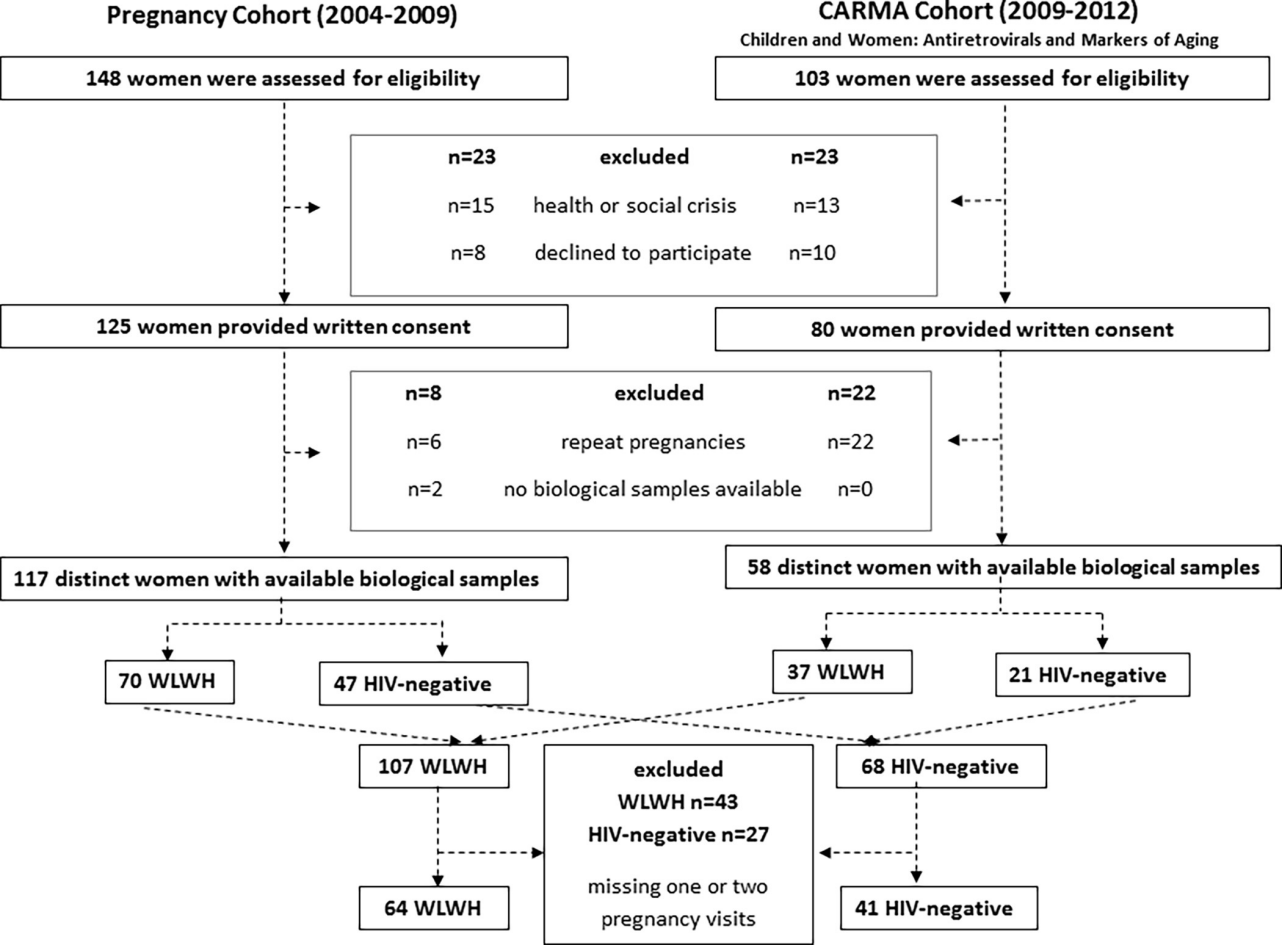
Schematic of study participants.

The original participation rate in the cohorts was high (>90%). Of note, among the 251 pregnancies in 223 women, after excluding repeat pregnancies and participants who missed one or more study visits in pregnancy, a total of 105 women (64 WLWH and 41 HIV-negative) were included in this study (Fig 1).

Generally, there were few differences between women who were included *vs*. excluded in this analysis (S1 Table and S2 Table). WLWH who were included had longer duration of HIV infection and longer exposure to cART during the current pregnancy. WLWH who were included were less likely to have a low income, and to smoke throughout pregnancy. Importantly, there was no significant difference between women who were included *vs*. those who were excluded with respect to LTL at each visit. Table 1 presents the demographic, clinical, and environmental characteristics of the study participants.

**Table 1:** Demographic, clinical and environmental characteristics of study participants.

Characteristics	All women (n = 105)	WLWH (n = 64)	HIV-negative women (n = 41)	P value
**Maternal age at delivery (years)**	31 ± 6 (17–41)	31 ± 6 (17–41)	32 ± 5 (21–41)	0.45
**Mode of delivery**				
Vaginal	74 (70)	41 (64)	33 (80)	0.52
Non-emergency C-section	8 (8)	6 (9)	2 (5)	
Emergency C-section ^a^	23 (22)	17 (27)	6 (15)	
**Weeks of gestation at visit**				
A (n = 105, 64, 41)	19 ± 2 (13–23)	19 ± 2 (14–23)	19 ± 2 (13–22)	0.35
B (n = 105, 64, 41)	26 ± 2 (23–30)	26 ± 2 (23–30)	26 ± 1 (24–30)	0.71
C (n = 103, 62, 41)	34 ± 2 (30–40)	34 ± 2 (30–37)	35 ± 2 (32–40)	**0.02**
Del (n = 55, 55, n/a)	38 ± 2 (32–41)	38 ± 2 (32–41)	n/a	—
**GA at delivery (weeks)** **(n = 105, 64, 41)**	39 ± 2 (32–42)	38 ± 2 (32–42)	39 ± 2 (35–42)	0.17
**Preterm delivery (<37 weeks)**	17 (16)	12 (19)	5 (12)	0.37
**Infant SGA**^b^ **(n = 103, 62, 41)**	15 (15)	11 (18)	4 (10)	0.27
**Race/Ethnicity**				**<0.0001**
Indigenous/First Nations	26 (25)	23 (36)	3 (7)	
Black/African Canadians	11 (10)	11 (17)	0 (0)	
White/Caucasian	49 (47)	18 (28)	31 (76)	
Asian/Other	19 (18)	12 (19)	7 (17)	
**Income <$15,000/year**	44 (42)	33 (52)	11 (27)	**0.01**
**History of HCV infection** ^c^	27 (26)	25 (40)	2 (5)	**<0.0001**
HCV+ Antibody (n = 77, 60, 17)	25 (32)	23 (38)	2 (12)	**0.04**
**Substance use throughout pregnancy** ^d^				
Smoking ^e^	43 (41)	33 (52)	10 (24)	**0.006**
Illicit drug ^f^	13 (12)	9 (14)	4 (10)	0.51
Alcohol	8 (8)	3 (5)	5 (12)	0.16
**HIV-specific characteristics** ^g^				
Duration of HIV infection at delivery (years)	—	6.1 ± 4.5 (0.4–19.2)	—	—
Age at HIV diagnosis (years)	—	25 ± 6 (2–36)	—	—
CD4+ nadir (cells/μl)	—	288 ± 195 (10–910)	—	—
Log Highest HIV pVL in pregnancy	—	2.7 ± 1.1 (1.6–5.2)	—	—
Detectable HIV pVL at visit C (>50 copies/ml) (n = 63)		12 (19)		
Detectable HIV pVL at delivery (>50 copies/ml) (n = 45) ^h^	—	8 (18)	—	—
cART exposure during pregnancy (weeks)	—	25.1 ± 10.2 (0.3–41.7)	—	—
cART naïve pre-pregnancy	—	23 (36)	—	—
Conceived on cART	—	19 (30)	—	—
**On cART at visit**:				
A (n = 64)		34 (53)		
B (n = 64)		59 (92)		
C (n = 62) ^i^		62 (100)		
Del (n = 55)		55 (100)		
P-P (n = 59)		38 (64)		
Received PI/r-based regimen during pregnancy	—	39 (61)	—	—

Data are presented as mean ± SD (range) or n (% of total) unless otherwise indicated. Abbreviations: WLWH, women living with HIV; GA, gestational age; SGA; small for gestational age, HCV, Hepatitis C Virus; pVL, plasma viral load; cART, combination antiretroviral therapy; PI/r, ritonavir-boosted protease inhibitor. Characteristics were compared between groups using Fisher's exact test for categorical variables and Student t-test for continuous variables.

^a^ Mode of C-section is not known for WLWH (n = 1) and HIV-negative women (n = 1) and considered as emergency C-section.

^b^ SGA infants have birth weights below the 10^th^ percentile for an infant population of the same GA and sex. In this study, SGA is calculated according to the British Columbia (BC), Canada statistics provided by perinatal service BC.

^c^ History of HCV infection was defined as self-report of HCV+ status and/or a lab test result.

^d^ Substance use throughout pregnancy is defined as self-reported use of substance at ≥3 visits during pregnancy inclusive of the period prior to delivery.

^e^ Smoking includes tobacco and/or marijuana and 8/9 participants who self-reported marijuana use throughout pregnancy reported tobacco use as well.

^f^ Illicit drug includes heroin, cocaine, opioids, amphetamines, benzodiazepenes and/or 3, 4-methylenedioxy-methamphetamine (MDMA).

^g^ Data was imputed for a total of 6 WLWH: CD4+ count and HIV pVL at visit A (n = 2), visit C (n = 1), CD4+ count (n = 2) and pVL (n = 1) at visit B.

^h^ Of note, 14 out of 19 women with unavailable HIV pVL at delivery, achieved undetectable HIV pVL at visit C. Considering available data at visit C and delivery visit, 51 out 59 women for whom pVL data was available (86%) had undetectable HIV pVL at the end of their pregnancy and/or delivery.

^i^ Blood specimens collected at delivery visit were used in the analyses for two WLWH for whom no visit C blood specimen was collected. The weeks of gestation at delivery for one woman was 32 and for the other one was 40, both within the visit C range.

The two groups were similar with respect to age, mode of delivery, weeks of gestation at first and second study visit, and delivery visit, rate of preterm delivery, and rate of infant small for gestational age (SGA). However, race/ethnicity was different between the two groups, with no Black/African Canadians and significantly fewer Indigenous/First Nations in the control group. WLWH were also more likely to have a history of HCV infection, and to have a low income compared to the control group. Both groups showed similar rates of substance and alcohol use throughout pregnancy but WLWH were more likely to smoke throughout pregnancy than HIV-negative women. The rates of substance use at each visit are presented in the S1 Fig.

**Fig 2:**
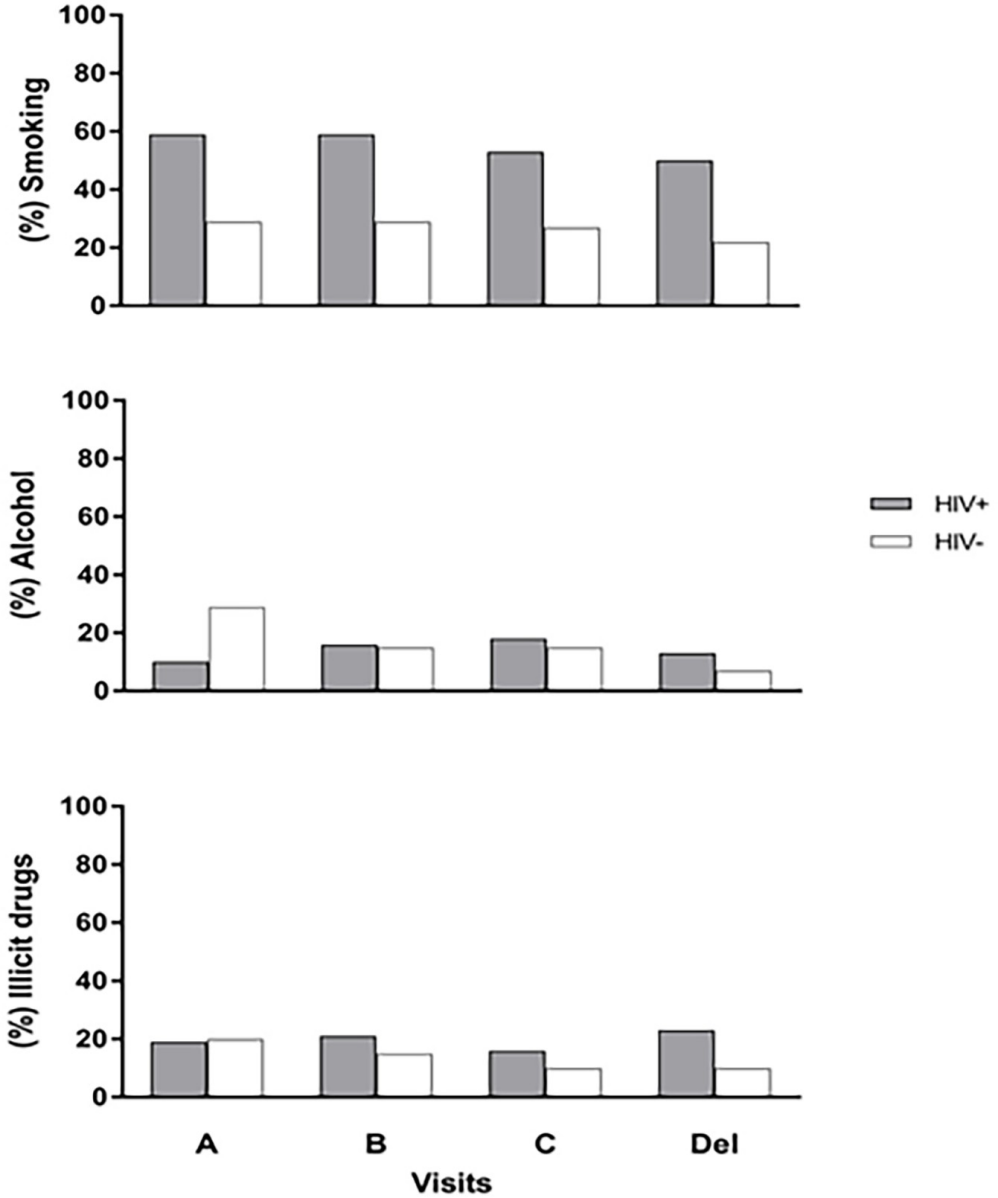
HIV specific characteristics at visit. A) % with detectable HIV plasma viral load (pVL) (> 50 copies/ml) vs. non-detectable, (B) cART status (% on vs. off cART), (C) CD4 count (cells/μl), (D) Log HIV pVL.

Within WLWH, the time since HIV diagnosis ranged from very recent (likely diagnosed during routine HIV testing in pregnancy) to almost two decades. Approximately 30% of WLWH conceived on cART, and 36% were cART-naïve prior to their pregnancy. Additionally, 34% were cART experienced before this pregnancy but were not on cART at the time of conception. All WLWH were on cART by their third visit. Considering available data at visit C and delivery visit, 51 out 59 (86%) women for whom pVL data was available had undetectable HIV pVL at the end of their pregnancy and/or delivery. More than half (64%) continued cART post-partum, as per standard of care at the time. HIV specific characteristics at visit are presented in Fig 2.

The cART regimens varied according to treatment guidelines/standard of care at the time, as well as individual circumstances (S3 Table). Thirty-nine (61%) WLWH received a PI/r-based regimen. The most common NRTI backbone was zidovudine (AZT)/lamivudine (3TC) (n = 47) combined with a PI; ritonavir-boosted lopinavir (LPV/r) (n = 27) or nelfinavir (NFV) (n = 19).

### Univariable association with LTL

In the univariable MANOVA analyses of all women (Table 2), HIV+ status, history of HCV infection, and smoking throughout pregnancy were significantly associated with shorter LTL.

**Table 2:** MANOVA models investigating the univariable association of predictors that may affect relative LTL in all women and separated by HIV status.

Predictors	All women (n = 105)			WLWH (n = 64)			HIV-negative women (n = 41)		
	β	95%CI	P Value	β	95% CI	P Value	β	95%CI	P Value
**Maternal age at delivery (per year)**	0.02	-0.07–0.11	0.66	0.06	-0.04–0.16	0.25	-0.09	-0.25–0.07	0.27
Maternal age at delivery*weeks of gestation	-0.02	-0.03 –-0.01	**0.002**	-0.01	-0.02 –-0.00	**0.02**	-0.03	-0.07–0.01	0.08
**Living with HIV** **(yes *vs*. no)**	-0.50	-0.01 –-0.99	**0.04**					—	—
**Weeks of gestation** **at visit**									
A	-0.05	-0.13–0.04	0.26	0.02	0.08–0.12	0.71	-0.19	-0.33 –-0.05	**0.008**
B	0.10	-0.02–0.22	0.10	0.09	-0.06–0.24	0.24	0.09	-0.13–0.30	0.42
C	0.02	-0.08–0.12	0.12	0.01	-0.12–0.15	0.82	-0.04	-0.23–0.15	0.67
**Preterm delivery (GA<37 weeks)**	-0.28	-0.95–0.37	0.39	0.45	-1.24–0.32	0.24	-0.10	-1.32–1.12	0.87
**Race/Ethnicity** **(Ref: Caucasian)**									
Indigenous/First Nations	-0.62	-1.51–0.26	0.37	-0.51	-1.44–0.42	0.08	0.12	-2.00–2.24	0.96
Black/African Canadians	0.00	-1.20–1.19		0.27	-0.91–1.46		—	—	
Asian/other	0.71	-0.26–1.69		1.22	0.08–2.37		-0.20	-1.87–1.47	
**Income <$15,000/year** **(yes *vs*. no)**	-0.19	-0.68–0.30	0.44	-0.21	-0.82–0.40	0.49	0.20	-0.70–1.10	0.66
**History of HCV infection** ^a^ **(yes *vs*. no)**	-0.54	-1.09–0.0	0.05	-0.24	-0.87–0.39	0.45	-1.56	-3.34–0.23	0.09
**Substance use throughout pregnancy** ^b^ **(yes *vs*. no)**									
Smoking ^c^	-0.68	-1.56 –-0.20	**0.006**	-0.70	-1.29 –-0.11	0.02	-0.34	-1.27–0.58	0.45
Illicit drug ^d^	0.09	-0.65–0.83	0.80	0.08	-0.80–0.97	0.85	0.26	-1.08–1.60	0.70
Alcohol	0.43	-0.48–1.34	0.35	1.36	-0.05–2.77	0.06	-0.38	-1.59–0.83	0.53
**Cohort (CARMA *vs*. Pregnancy)**	-0.28	-0.80–0.24	0.24	-0.75	-1.36 –-0.14	**0.02**	0.82	-0.07–1.71	0.07
**Received PI/r based regimen during pregnancy (yes *vs*. no)**	—	—	—	-0.70	-1.31 –-0.09	**0.03**	—	—	—
**CD4+ (cells/𝛍l) at visit**									
A	—	—	—	0.00	-0.00–0.00	0.56	—	—	—
B	—	—	—	-0.00	-0.00–0.00	0.61	—	—	—
C	—	—	—	0.00	-0.00–0.00	0.78	—	—	—
**Detectable HIV pVL at visit (>50 copies/ml) (yes *vs*. no)**									
A	—	—	—	-0.10	-0.30–0.10	0.33	—	—	—
B	—	—	—	-0.13	-0.37–0.11	0.29	—	—	—
C	—	—	—	-0.19	-0.48–0.10	0.20	—	—	—
**cART status at visit (yes *vs*. no)**									
A	—	—	—	0.02	-0.18–0.22	0.86	—	—	—
B	—	—	—	-0.20	-0.64–0.24	0.37	—	—	—

Abbreviations: WLWH, women living with HIV; GA, gestational age; HCV, Hepatitis C Virus; CD4+, cluster of differentiation 4; pVL, plasma viral load; cART, combination antiretroviral therapy

^a^ History of HCV infection was defined as self-report of HCV+ status and/or a lab test result.

^b^ Substance use throughout pregnancy is defined as self-reported use of substance at ≥3 visits during pregnancy inclusive of the period prior to delivery.

^c^ Smoking includes tobacco and/or marijuana and 8/9 participants who self-reported marijuana use throughout pregnancy reported tobacco use as well.

^d^ Illicit drug includes heroin, cocaine, opioids, amphetamines, benzodiazepenes and/or 3, 4-methylenedioxy-methamphetamine (MDMA).

**Fig 3:**
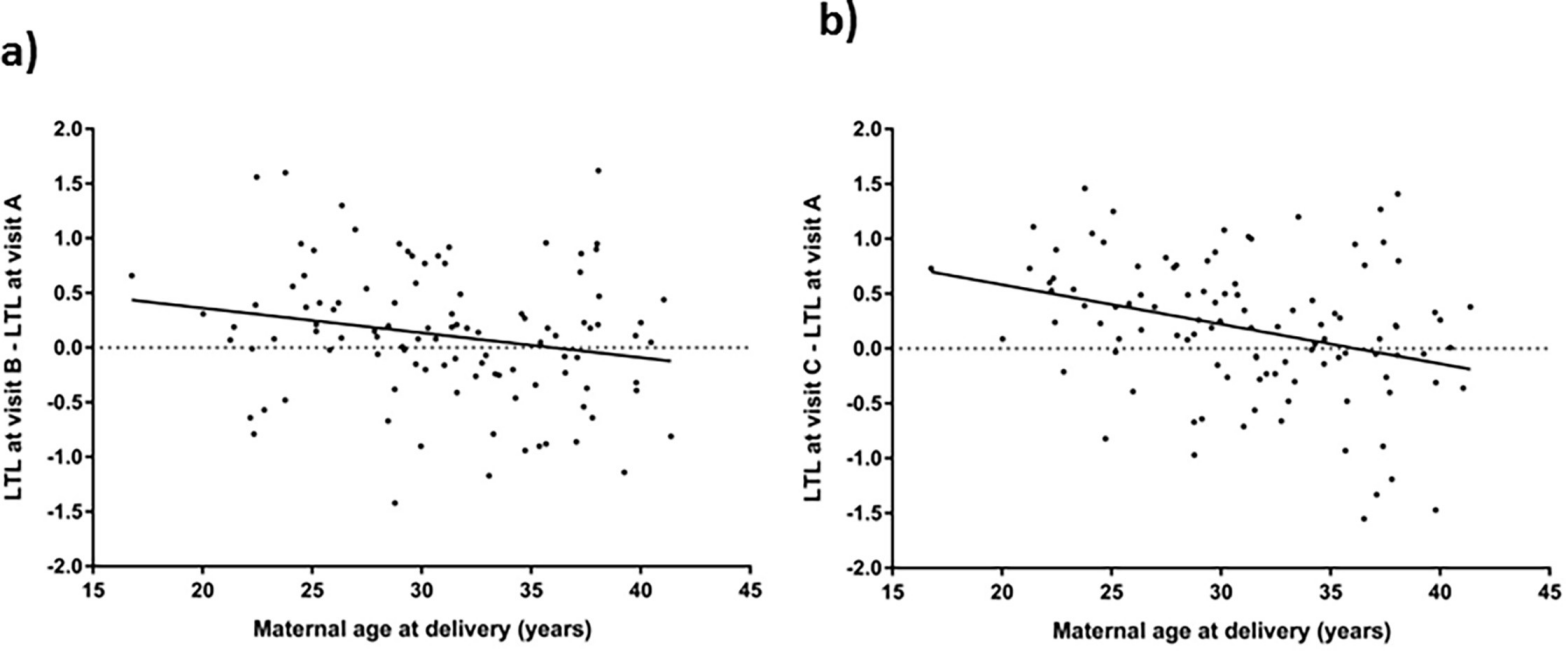
Linear regression illustrating the interaction between maternal age at delivery and change in LTL between visits. Change between visits A and B (a) and visits C and A (b).

All other variables considered, including maternal age, weeks of gestation at visit, preterm delivery, race/ethnicity, income, cohort, alcohol, and substance use (or use of substances of addiction) throughout pregnancy showed no association with LTL. Upon examining variables within subjects, a significant interaction was noted between maternal age and weeks of gestation with respect to LTL among all women (p = 0.002) and WLWH (p = 0.02). Fig 3 illustrates this statistical interaction whereby apparent LTL lengthening is seen as pregnancy progresses, more so in women younger than 35 years old.

**Fig 4:**
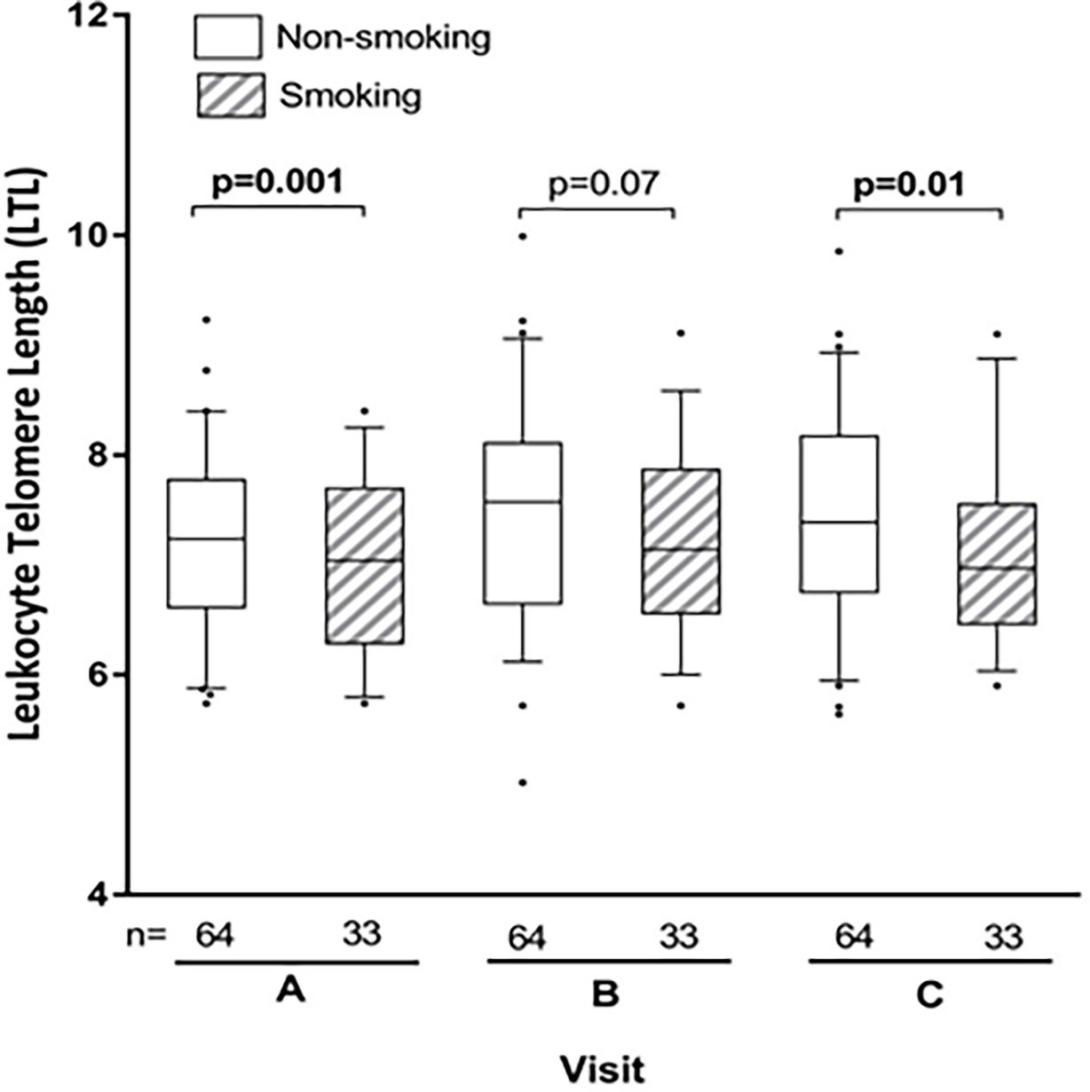
Relative LTL at visit dichotomized according to smoking status throughout pregnancy in all participants. Smoking women self-reported smoking throughout pregnancy (at ≥3 visits during pregnancy inclusive of the period prior to delivery). Comparisons between groups used Student's t-test.

Univariable MANOVA analysis across the visits suggested an association between smoking (defined as self-reporting smoking at ≥3 visits during pregnancy inclusive of the period prior to delivery) and shorter LTL among all (β = -0.70) participants and WLWH (β = -0.68), but not among the HIV-negative group. As the mean LTL for the entire study sample at visit A was 7.4 ± 0.9, this represents a difference of approximately 10% in LTL. Fig 4 further illustrates that cross-sectionally, smokers have shorter LTL at each visit (visit A, p = 0.001; visit B, p = 0.07; visit C, p = 0.01).

Given that these results pointed toward an effect of smoking that was at least as important as our initially hypothesized HIV effect, our study participants were re-examined based on their smoking status. Women who smoked throughout pregnancy were younger, delivered at an earlier GA, were significantly more likely to be living with HIV, to have a low income, a history of HCV infection, and report using one or more substances throughout pregnancy (S4 Table). Of note, 30% of women who smoked throughout pregnancy also self-reported substance use, while none of the non-smokers did.

**Fig 5:**
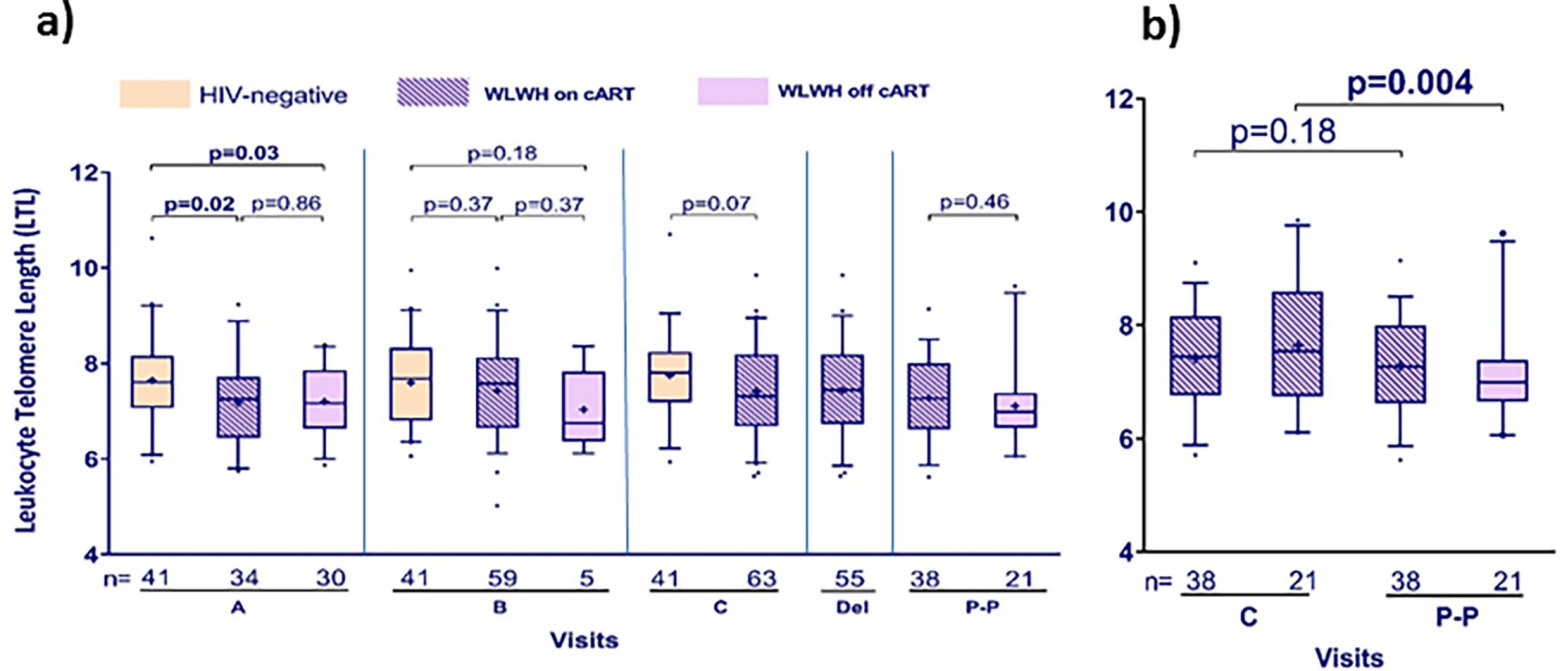
Relative LTL at visit dichotomized according to HIV/cART status. Comparisons between groups (a) used Student's t-test, and comparisons within WLWH at visit C and P-P (b) used paired t-test.

The univariable MANOVA analysis among WLWH women suggested that being a CARMA cohort participant and receiving a PI/r regimen during pregnancy were also associated with shorter LTL (Table 2). In contrast, the reverse was observed for HIV-negative women, whereby being a Pregnancy cohort participant was the strongest predictor of shorter LTL (p = 0.07), apart from the weeks of gestation at visit A. This observation prompted us to examine the makeup and distribution of cART type in each cohort. As shown in S5 Table, collinearity existed between cohort and cART type, whereby WLWH in the CARMA cohort were more likely to receive a PI/r regimen (91%) than WLWH enrolled in the earlier Pregnancy cohort (32%) (p<0.001); they also had shorter LTL. This difference in cART usage between the two cohorts, mostly driven by change in prescribing practices over time, likely explains the cohort association in WLWH. There was no significant difference between the characteristics of the HIV-negative women in the CARMA and Pregnancy cohorts. However, the rate of smoking in the Pregnancy cohort was 29% while that in the CARMA cohort was 10% and only 24% of the HIV-negative women were from the CARMA cohort. No significant relationships were seen between LTL and either CD4+ cell counts, detectable HIV pVL, or cART status at visit. In Fig 5, LTL at each visit is presented (cross-sectional), dichotomized according to HIV/cART status.

As noted above, there is no LTL difference between WLWH who were on cART *vs*. off cART at visit A and B. Among 25 women who initiated cART between visits A and B, LTL at visit B was non-significantly longer (p = 0.06) than at visit A, with no evidence suggesting LTL attrition upon cART initiation. HIV-negative women had longer LTL than WLWH at visit A, and this difference waned later in pregnancy (S6 Table). Among WLWH with a post-partum visit, women who stopped cART after delivery had significantly shorter LTL post-partum than at visit C, while LTL did not change for those who remained on cART (Fig 5B).

### Multivariable associations with LTL

**Fig 6:**
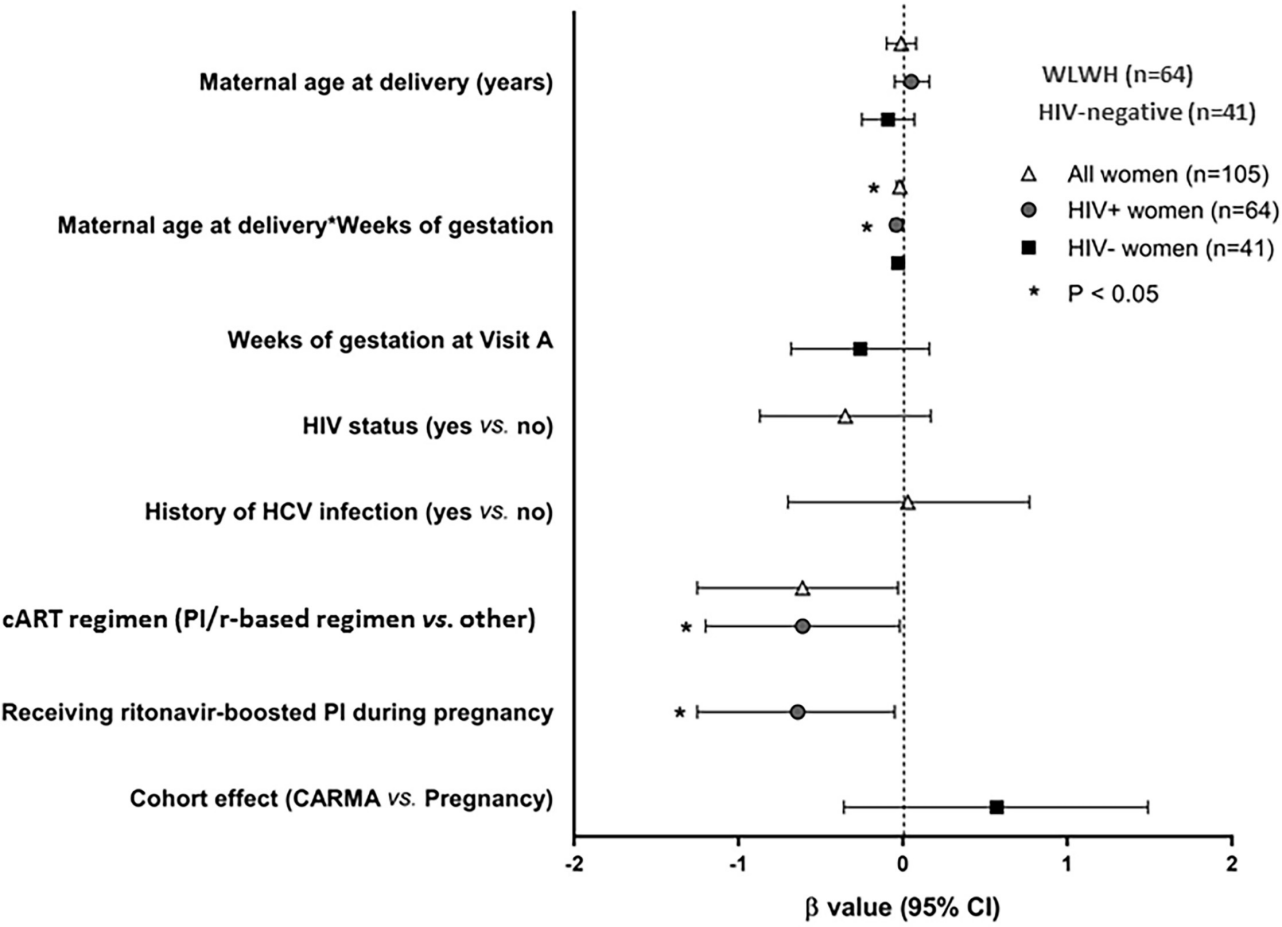
Forrest plot of the multivariable analyses of the associations with relative LTL. MANOVA models were separated by group: all women (light gray triangle), WLWH (dark gray circle), and HIV-negative women (black square). β and 95% confidence intervals (CI) are shown; negative β values indicate associations with shorter LTL. Abbreviations: HCV, Hepatitis C Virus; PI/r, ritonavir-boosted protease inhibitor. ^a^ History of HCV infection is based on lab results and/or self-report. ^b^ Smoking throughout pregnancy is defined as self-reported use of tobacco and/or marijuana at ≥3 visits inclusive of the period prior to delivery. Only variable with p≤0.1 univariately are shown.

In the two multivariable MANOVA models for all women and for WLWH (Fig 6), the strongest predictor of shorter LTL was the interaction between maternal age and weeks of gestation at visit (p = 0.01, β = -0.02 and p = 0.01, β = -0.04, respectively). This suggests that the phenomenon of apparent longer LTL as pregnancy progresses is more pronounced among younger women, as seen in the univariable model.

Smoking throughout pregnancy was the second strongest predictor of shorter LTL among all women (p = 0.06) with the largest effect size (β = -0.61). However, this association only reached statistical significance in the WLWH group (p = 0.04). In addition, having received a PI/r showed an independent association with shorter LTL (p = 0.03), and a similar effect size (β = -0.64). For the HIV-negative group, none of the possible predictors examined showed an independent association with LTL (Fig 6, S7 Table).

## Discussion

This study is the first to report trans-pregnancy data on LTL. In this cohort, both WLWH and HIV-negative women showed similar rates of preterm delivery that were higher than those reported in Canada (7.9%) [47] and British Columbia (9.7%) [48]. This is in contrast to previous studies, which reported an increased incidence of preterm delivery among WLWH treated with cART compared to HIV-negative women [49, 50]. This is likely explained by the fact that our HIV-negative participants were deliberately targeted for enrolment. Consequently, they shared many non-HIV related risk factors for preterm delivery with WLWH [51–53], which is not the case in other cohort studies reported in the literature. Shorter LTL was not associated with preterm delivery in this study.

In high resource countries, smoking is highly prevalent among HIV populations [54, 55]. In agreement with this, we observed a rate of smoking noticeably higher (43%) than that reported for Canadian pregnant women (17%) [56]. This is likely influenced by the socio-economic status and ethnic makeup of our cohort [57]. Our data also showed no association between maternal age and shorter LTL. This may in part be related to the fact that most women were within a narrow age range (25 to 35 years), an age span during which LTL is more stable than earlier or later in life [58].

The dynamics of LTL during pregnancy have not been previously studied longitudinally. We demonstrated that, within woman, LTL at three visits are significantly correlated throughout pregnancy, and relatively stable. Our data further suggest that relative LTL appear to lengthen as pregnancy progresses. This may be related to increases in blood volume triggering the production of new leukocytes with longer LTL. The fact that this effect was most pronounced among younger women could be explained by age-related differences in leukocyte turnover and/or hormone levels during pregnancy.

Although both living with HIV and smoking throughout pregnancy showed a univariate association with shorter LTL, and of similar effect size, in the multivariable model, LTL was most influenced by pregnancy progression. In the latter model, the effect of both HIV and smoking was in the expected direction but did not reach significance. In the WLWH multivariable model however, smoking remained independently associated with shorter LTL, but this association may be confounded by other substances of addiction. This observation is in agreement with previous studies reporting the deleterious effects of smoking and illicit drugs on LTL, including one in mothers and their infants [37, 59–65]. Increased oxidative stress, leukocyte turnover, and apoptosis as well as modulation of gene expression have been linked shorter LTL in smokers [66, 67]. Furthermore, the above-mentioned interaction whereby younger women experienced greater LTL gain as pregnancy progressed may also have been influenced by smoking or rather changes in smoking behavior. Indeed, in our study younger women were more likely to smoke. Given the stigma associated with smoking during pregnancy, it is possible that some participants reduced the intensity of their smoking, which could positively impact LTL. Because we did not capture data on smoking intensity, we can only speculate about this. Smoking is a known risk factor for adverse pregnancy outcomes [68–70] and infants born to women who smoked were born at an earlier GA. Taken together, our findings further support the importance of smoking cessation in pregnancy.

Among WLWH, receiving a PI/r-based cART regimen was also associated with shorter LTL. Although *in vitro* and *ex vivo* studies have shown telomerase inhibition by NRTIs [28, 29], a recent study of virally controlled PLWH randomized to receive ritonavir-boosted darunavir with or without two NRTIs reported no difference in peripheral blood mononucleated cell (PBMC) telomere length between groups, nor was it changed upon cessation of the NRTIs [71]. Consistent with our general observation that being on cART may positively affect LTL, a study recently reported increases in LTL upon initiating cART [40]. PIs have been shown to increase oxidative stress in a cell culture model [72], something that could play a role here. Some studies report increased risk of preterm delivery among women receiving PI/r [73–77], but others do not [78, 79]. Our study was not powered to examine the relationships with preterm delivery. The observed association between PI/r and shorter LTL requires further study.

Among WLWH, the shorter LTL observed post-partum may be related to cART interruption although other factors such as changes in leukocyte count [80] may also play a role. Our data suggest that staying on cART may protect LTL, possibly through reduced immune activation hence oxidative stress.

### Strengths and limitations

The major strength of this study was the longitudinal design, which allowed insight into the dynamics of LTL during pregnancy. Furthermore, the study and control groups were well balanced with respect to important factors such as age, illicit drugs, and alcohol use throughout pregnancy, reducing the effect of these potential confounders. However, this study also has several limitations. We measured TL in total leukocytes and are not able to address TL change in specific cell subsets [81]. Although it has been shown that the count of various cell subsets varies during pregnancy, their proportion remains largely unchanged [82]. Further, TL within cell subsets were shown to be highly correlated to that of PBMC TL in a study of women participants [83]. Hormone levels were not measured in our study. We therefore cannot ascertain the possible effect of hormone level changes on LTL. Specifically, estrogen which increases during pregnancy and decreases post-partum, has been associated with increased telomerase activity and antioxidant effects [84–88]. Our data could be at least partially explained by expected estrogen changes, although we also cannot address this. Moreover, we could not ascertain whether the decrease in LTL seen post-partum in WLWH is related to pregnancy ending or is specific to this population. Older maternal age at the time of last child’s birth has been associated with longer LTL [89]. Because our participants were still in their reproductive years, and since many had subsequent pregnancies, we could not consider this variable. Finally, the groups were not well balanced with respect to ethnic makeup, income, and history of HCV infection and these remain possible confounders for the comparisons between women living with HIV and HIV-negative women. However, these factors would have little influence on the within-woman longitudinal observations. Although we have indicated that the self-reported smoking data are robust, the frequency, intensity, or timing of smoking were only available for a subset of participants, hence were not considered. However, based on the participants for whom these details were known, smoking habits did not change noticeably over time. Overall, the HIV-negative model explained 40% of the LTL variance, which is higher than for WLWH and all participants’ models, suggesting that we may be missing important yet unidentified factors.

In conclusion, there were no significant differences in pregnancy outcomes between WLWH and HIV-negative women in this study. This may be explained by the fact that our groups were well similar with respect to non-HIV risk factors for preterm delivery, as these were high in both groups, and may have minimized differences in LTL and adverse pregnancy outcomes between our groups. Smoking throughout pregnancy and receiving a PI/r-based regimen were independently associated with shorter LTL among pregnant WLWH. Whether these reflect telomere attrition or redistribution of cellular subsets remains unclear. Pending confirmation in an independent cohort, our study suggests that modifiable factors such as smoking may exert as much if not more influence on LTL than HIV-related factors.

## Supporting information

S1 Supporting information File name: Supporting information text. (DOCX)

S1 Table Comparison of demographic, clinical and environmental characteristics between WLWH who were included in and excluded from the analyses. File name: S1 Table.
(DOCX)

S2 Table Comparison of demographic, clinical, and environmental characteristics between HIV-negative women who were included in and excluded from the analyses. File name: S2 Table.
(DOCX)

S3 Table Combination antiretroviral therapy (cART) regimens taken during pregnancy by WLWH (n = 64). File name: S3 Table.
(DOCX)

S4 Table Demographic and clinical characteristics of study participants separated by smoking status. File name: S4 Table.
(DOCX)

S5 Table Demographic, clinical, and laboratory characteristics of study participants separated by cohort. File name: S5 Table.
(DOCX)

S6 Table Leukocyte telomere length (LTL) at visit A, B, and C separated by HIV status and at delivery and post-partum only for WLWH.
(DOCX)

S7 Table Multivariate analyses of the association between various factors and LTL in all participants and separated by HIV status. File name: S7 Table.
(DOCX)

S1 Fig Rate of substance use at each visit. (DOCX)
