# Supplementary material for: Dynamics of leukocyte telomere length in pregnant women living with HIV, and HIV-negative pregnant women: A longitudinal observational study
Source: PLoS One. 2019 Mar 6;14(3):e0212273. doi: 10.1371/journal.pone.0212273 (PMC6402636; doi:10.1371/journal.pone.0212273)
Supplement: S1 Supporting information — (DOCX) [file pone.0212273.s001.docx]

**Supporting information**

**Supporting text**

**Description of variables**

Age, ethnicity, income, and substance use history (smoking, alcohol, and illicit drugs) were obtained from the participants through self-report. Due to some heterogeneity in data collection between pregnancy and CARMA cohorts, we elected to code substance use at visit as a yes/no, irrespective of intensity. Substance use (smoking, alcohol, illicit drug use) throughout pregnancy was defined as self-reported use of substance at ≥3 visits during pregnancy inclusive of the period prior to delivery. Non-users were all those self-reporting use at <3 visits during pregnancy; only 7 women reported smoking at only 1 or 2 visits. Smoking was defined as tobacco and/or marijuana consumption. Marijuana use was highly collinear with tobacco use (89% of marijuana users also used tobacco) in this study. For this reason, we elected to combine both in the smoking variable.

Most cohort studies collect and use self-report data for smoking. However, due to stigma associated with smoking during pregnancy, there is a risk that the behavior may be underreported, leading to study biases. In another study (manuscript submitted), we examined the reliability of self-reported smoking (tobacco use) during pregnancy in our cohorts by measuring plasma cotinine concentration and determining the relationship between the two measures. We found excellent concordance (91%) between plasma cotinine and smoking self-report, indicating that our smoking data are reliable as a surrogate for tobacco exposure. Illicit drugs include heroin, cocaine, opioids, amphetamines, benzodiazepenes and/or 3, 4-methylenedioxy-methamphetamine (MDMA). Smoking and use of illicit drug were collinear and the all women who used illicit drugs also smoked throughout pregnancy.

History of HCV infection was defined as self-report of HCV+ status and/or a lab test result. The HCV antibody tests were part of early pregnancy testing and results were available for the majority of WLWH through their medical charts. Results of HCV antibody test were available for total of 77 participants (WLWH, n=60; HIV-negative, n=17).

For WLWH, date of HIV diagnosis, CD4+ nadir, CD4+ count at visit, HIV pVL at visit, cART status at visit (on *vs.* off), being antiretroviral therapy naïve, having conceived on cART, duration of cART in pregnancy (weeks), type of cART regimens during pregnancy were obtained. cART regimens were categorized as containing a ritonavir-boosted PI or not. The detail of cART regimens with the longest exposure time during pregnancy is presented in eTable 4. In case of missing CD4+ count or HIV pVL at pregnancy visits, we imputed the data. When the missing value was for a visit between 2 other visits (i.e. visit B or Visit C), we used the average of the 2 other available values. To impute the data for visit A, we carried backward the next available value (values from visit B). Taken together, we imputed data for a total of 6 WLWH: both CD4 count and HIV pVL at visit A (n=2) and visit C (n=1), CD4 count (n=2) and pVL (n=1) at visit B.

Preterm birth is defined as childbirth occurring at gestational age (GA) less than 37 weeks^1^. Infant small for gestational age (SGA) have birth weights below the 10^th^ percentile for infants of the same GA and sex. In this study, infant SGA was calculated according to the British Columbia statistics provided by perinatal service BC.

**Relative LTL measurement: qPCR assay and quality control**

Whole blood DNA was extracted using the QIAamp DNA Mini Kit (Qiagen) on the QIAcube, according to the manufacturer’s “Blood and Body Fluid” protocol with the following modifications: 0.1 ml of blood was diluted 1:1 with 0.1 ml phosphate buffered saline prior to extraction, and the DNA was eluted into a final volume of 0.1 ml of buffer AE (Qiagen). Relative LTL were measured by a monochromatic multiplex qPCR assay modified from^2^ and described in detail previously^3^. Briefly, human albumin was used as single copy nuclear gene and the assays were done on a LightCycler® 480 (Roche). Each 10 µL reaction mix contained 2 µl of DNA extract and 8 µL of 1X SYBR green master mix (Roche). The final concentrations of reagents in the master mix were 1X LightCycler® SYBR Green I Master (Roche), 1.2 mM EDTA (Sigma-Aldrich), and 0.9 µM of each of the four multiplex primers as previously described. A standard curve was generated by serial dilutions (1:2) of pooled human whole blood DNA ranging from ~45 500 to 360 copies of albumin single copy gene (S) and ~160 to 1 relative copies of Telomere (T). This corresponds to a total DNA concentration of 21 to 0.16 ng/µl. LTL measurements were done in duplicate and the LightCycler raw text files were converted to grid format using LC480 conversion free software developed by the Heart Failure Research Center in Amsterdam, the Netherlands ([http://www.hartfaalcentrum.nl/index.php? main=files&fileName=LC480Conversion.zip&​ description=LC480%20Conversion&sub=LC480​Conversion](http://www.hartfaalcentrum.nl/index.php?main=files&fileName=LC480Conversion.zip&description=LC480%20Conversion&sub=LC480Conversion)). The converted data were analyzed using LinRegPCR free software developed by Rutjer *et al*^4^. T and S copy numbers were calculated based on the standard curves. The relative LTL value were expressed as the average of the duplicate T*1000/S ratios (these were multiplied by 2.33 to calibrate according to lymphocyte TL values previously obtained by Flow-FISH^5^).

Duplicates with an absolute difference >15% were rejected and repeated. Each run contained 40 duplicates, and the mean absolute difference between replicates was<10%. Two internal controls (IC) were included in each run showed inter-run coefficients of variation (%CV: SD*100/mean) of 3.24% and 3.62% over 26 separate runs for LTL. For each run, the PCR efficiency and IC values had to lie within mean ± 2 SD, and the difference between the PCR efficiencies of both genes were kept <0.05.

**Supplementary References**

1. Mathews TJ, MacDorman MF. Infant mortality statistics from the 2003 period linked birth/infant death data set. Natl Vital Stat Rep. 2006;54(16): 1-29. https://www.ncbi.nlm.nih.gov/pubmed/16711376.
2. Cawthon RM. Telomere length measurement by a novel monochrome multiplex quantitative PCR method. Nucleic Acids Res. 2009;37(3). doi:10.1093/nar/gkn1027.
3. Hsieh AY, Saberi S, Ajaykumar A, et al. Optimization of a Relative Telomere Length Assay by Monochromatic Multiplex Real-Time Quantitative PCR on the LightCycler 480: Sources of Variability and Quality Control Considerations. J Mol Diagn. 2016;18(3):425-437. doi:10.1016/j.jmoldx.2016.01.004.
4. Ruijter JM, Ramakers C, Hoogaars WM, et al. Amplification efficiency: linking baseline and bias in the analysis of quantitative PCR data. Nucleic Acids Res. 2009;37(6):e45. doi:10.1093/nar/gkp045.
5. Imam T, Jitratkosol MHJ, Soudeyns H, et al. Leukocyte telomere length in HIV-infected pregnant women treated with antiretroviral drugs during pregnancy and their uninfected infants. J Acquir Immune Defic Syndr. 2012;60(5):495-502. doi:10.1097/QAI.0b013e31825aa89c.
