# Supplementary material for: Dynamics of leukocyte telomere length in pregnant women living with HIV, and HIV-negative pregnant women: A longitudinal observational study
Source: PLoS One. 2019 Mar 6;14(3):e0212273. doi: 10.1371/journal.pone.0212273 (PMC6402636; doi:10.1371/journal.pone.0212273)
Supplement: S1 Table — File name: S1 Table. (DOCX) [file pone.0212273.s002.docx]

**S1 Table.** Comparison of demographic, clinical and environmental characteristics between WLWH who were included in and excluded from the analyses.

| **Characteristics** | **WLWH included (n=64)** | **WLWH excluded (n=43)** | **P value** |
| --- | --- | --- | --- |
| **Maternal age at delivery (years)** | 31 ± 6 (17-41) | 31 ± 5 (18-45) | 0.76 |
| **Weeks of gestation at visit** |  |  |  |
| A (n=64, 14) | 19 ± 2 (14-23) | 20 ± 2 (16-23) | 0.14 |
| B (n=64, 30) | 26 ± 2 (23-30) | 27 ± 2 (23-31) | 0.13 |
| C (n=62, 31) | 34 ± 2 (30-37) | 34 ± 2 (31-38) | **0.04** |
| Del (n=55, 26) | 38 ± 2 (32-41) | 39 ± 2 (31-41) | 0.34 |
| **GA at delivery (weeks)** | 38 ± 2 (32-42) | 38 ± 3 (31-41) | 0.77 |
| **Preterm delivery (GA<37 weeks)** | 12 (19) | 9 (21) | 0.78 |
| **Race/Ethnicity (n=64, 42)** |  |  | 0.23 |
| Indigenous/First Nations | 23 (36) | 11 (26) |  |
| Black/African Canadians | 11 (17) | 6 (14) |  |
| White/ Caucasian | 18 (28) | 20 (48) |  |
| Asian/Other | 12 (19) | 5 (12) |  |
| **Income <$15,000/year (n=64, 41)** | 33 (52) | 22 (54) | 0.83 |
| **History of HCV infection** | 25 (39) | 16 (37) | 0.85 |
| **Substance use throughout pregnancy**^a^ |  |  |  |
| Smoking^b^ (n=64, 37) | 33 (52) | 15 (41) | 0.28 |
| Illicit drug^c^ (n=64, 35) | 9 (14) | 4 (11) | 0.71 |
| Alcohol (n=64, 35) | 3 (5) | 2 (6) | 0.82 |
| **HIV-specific characteristics** |  |  |  |
| Duration of HIV infection at delivery (years) | 6.1 ± 4.5 (0.4-19.2) | 4.4 ± 4.3 (0.2-17.1) | **0.03** |
| Age at HIV diagnosis (years) | 25 ± 6 (2-36) | 26 ± 5 (15-37) | 0.28 |
| CD4+ nadir (cells/µl) | 288 ± 195 (10-910) | 311 ± 210 (20-730) | 0.67 |
| Log Highest HIV pVL in pregnancy (n=60, 41) | 2.8 ± 1.1 (1.69-5.2) | 3.0 ± 1.1 (1.6-5.0) | 0.52 |
| Detectable HIV pVL at delivery (n=45, 20) | 9 (20) | 2 (10) | 0.32 |
| cART exposure during pregnancy (weeks) (n=64, 40) | 25.1 ± 10.2 (0.3-41.7) | 20.5 ± 10.8 (1.4-41.1) | **0.03** |
| ART naïve pre-pregnancy | 23 (36) | 22 (51) | 0.12 |
| Conceived on cART | 19 (30) | 9 (41) | 0.31 |
| Received PI/r-based regimen | 39 (61) | 27 (63) | 0.85 |
| **LTL at Visit** |  |  |  |
| A (n=64, 13) | 7.2 ± 0.8 (5.7-9.2) | 6.8 ± 1.3 (4.6-9.0) | 0.19 |
| B (n=64, 29) | 7.4 ± 0.9 (5.0-10.0) | 7.7 ± 1.1 (5.1-11.1) | 0.14 |
| C (n=64, 31) | 7.4 ± 0.9 (5.6-9.8) | 7.8 ± 1.0 (6.0-10.4) | 0.07 |
| Del (n=55, 26) | 7.2 ± 1.1 (5.5-11.0) | 7.6 ± 0.9 (5.4-9.2) | 0.07 |
| P-P (n=59, 30) | 7.2 ± 0.8 (5.6-9.6) | 7.5 ± 1.0 (5.9-10.2) | 0.20 |

Data are presented as mean ± SD (range) or n (% of total included or excluded); unless otherwise indicated. Abbreviations: WLWH, women living with HIV; Del, delivery; GA, gestational age; HCV, Hepatitis C Virus; CD4+; cluster of differentiation; pVL, plasma viral load; cART, combination antiretroviral therapy; LTL, Leukocyte Telomere Length; P-P, Post-Partum.^a^Substance use throughout pregnancy is defined as self-reported use of substance at ≥3 visits during pregnancy inclusive of the period prior to delivery. ^b^Smoking includes tobacco and/or marijuana use throughout pregnancy. ^c^Illicit drug includes heroin, cocaine, opioids, amphetamines, benzodiazepenes, and/or 3, 4 methylenedioxy-mehamphetamine (MDMA).
