# Supplementary material for: Dynamics of leukocyte telomere length in pregnant women living with HIV, and HIV-negative pregnant women: A longitudinal observational study
Source: PLoS One. 2019 Mar 6;14(3):e0212273. doi: 10.1371/journal.pone.0212273 (PMC6402636; doi:10.1371/journal.pone.0212273)
Supplement: S2 Table — File name: S2 Table. (DOCX) [file pone.0212273.s003.docx]

**S2 Table.** Comparison of demographic, clinical, and environmental characteristics between HIV-negative women who were included in and excluded from the analyses.

| **Characteristics** | **HIV-negative women included (n=41)** | **HIV-negative women excluded (n=27)** | **P value** |
| --- | --- | --- | --- |
| **Maternal age at delivery (years)** | 31 ± 5 (21-41) | 31 ± 5 (21-43) | 0.63 |
| **Weeks of gestation at visit** |  |  |  |
| A (n=41, 13) | 19 ± 2 (13-22) | 19 ± 2 (16-23) | 0.37 |
| B (n=41, 17) | 26 ± 1 (24-30) | 27 ± 2 (23-30) | 0.50 |
| C (n=41, 19) | 35 ± 2 (32-40) | 34 ± 2 (32-38) | 0.49 |
| **GA at delivery** **(weeks)** | 40 ± 2 (35-42) | 38 ± 3 (29-41) | 0.20 |
| **Preterm delivery (GA<37 weeks)** | 5 (12) | 5 (19) | 0.47 |
| **Race/Ethnicity** |  |  | 0.08 |
| Indigenous/First Nations | 3 (7) | 7 (26) |  |
| Black/African Canadians | 0 (0) | 1 (4) |  |
| White/ Caucasian | 31 (76) | 14 (52) |  |
| Asian/Other | 7 (17) | 5 (18) |  |
| **Income <$15,000/year** | 11 (27) | 17 (63) | **0.003** |
| **History of HCV infection** | 2 (5) | 5 (19) | 0.07 |
| **Substance use throughout pregnancy (n=41, n=22)**^a^ |  |  |  |
| Smoking^b^ | 10 (24) | 13 (59) | **0.04** |
| Illicit drug^c^ | 4 (10) | 6 (27) | 0.16 |
| Alcohol | 5 (12) | 2 (9) | 0.52 |
| **LTL at Visit** |  |  |  |
| A (n=41, 14) | 7.6 ± 0.9 (6.0-10.6) | 7.4 ± 1.5 (5.8-10.2) | 0.54 |
| B (n=41, 18) | 7.6 ± 0.9 (6.0-9.9) | 7.7 ± 1.2 (6.1-10.2) | 0.63 |
| C (n=41, 19) | 7.7 ± 0.9 (5.9-10.7) | 7.9 ± 0.9 (6.5-9.9) | 0.54 |

Data are presented as mean ± SD (range) or n (% of total included or excluded); unless otherwise indicated.

Abbreviations: Del, delivery; GA, gestational age; HCV, Hepatitis C Virus; LTL, Leukocyte Telomere Length.

^a^ Substance use throughout pregnancy is defined as self-reported use of substance at ≥3 visits during pregnancy

inclusive of the period prior to delivery. ^b^Smoking includes tobacco and/or marijuana use throughout pregnancy.

^c^Illicit drug includes heroin, cocaine, opioids, amphetamines, benzodiazepenes and/or 3, 4-methylenedioxy-

methamphetamine (MDMA).
