# Supplementary material for: Dynamics of leukocyte telomere length in pregnant women living with HIV, and HIV-negative pregnant women: A longitudinal observational study
Source: PLoS One. 2019 Mar 6;14(3):e0212273. doi: 10.1371/journal.pone.0212273 (PMC6402636; doi:10.1371/journal.pone.0212273)
Supplement: S3 Table — File name: S3 Table. (DOCX) [file pone.0212273.s004.docx]

**S3 Table.** Combination antiretroviral therapy (cART) regimens taken during pregnancy by WLWH (n=64).

| **cART regimens** | **Base** | | **Back-bone** | |
| --- | --- | --- | --- | --- |
| **Ritonavir-boosted PI-based Regimens (n=39)** | | | | |
|  | **LPV/r** | 33 | AZT/3TC | 27 |
|  |  |  | ABC/3TC | 1 |
|  |  |  | TDF/FTC | 1 |
|  |  |  | D4T/3TC | 2 |
|  |  |  | AZT/ABC/3TC | 1 |
|  |  |  | AZT/ddI/3TC | 1 |
|  | **ATV/r** | 6 | ABC/3TC | 4 |
|  |  |  | TDF/FTC | 2 |
| **Other Regimens (n=25)** | | | | |
|  | **NFV** | 19 | AZT/3TC | 18 |
|  |  |  | D4T/3TC | 1 |
|  | **NVP** | 3 | AZT/3TC | 1 |
|  |  |  | ABC/3TC | 2 |
|  | **ATV** | 1 | TDF/3TC | 1 |
|  | **RTV** | 1 | AZT/3TC/TDF | 1 |
|  | --- | 1 | AZT/ABC/3TC | 1 |

PI, protease inhibitors; ABC, abacavir; AZT, zidovudine; 3TC, lamivudine; TDF, tenofovir; ddI, didanosine; d4T,
