# Supplementary material for: Dynamics of leukocyte telomere length in pregnant women living with HIV, and HIV-negative pregnant women: A longitudinal observational study
Source: PLoS One. 2019 Mar 6;14(3):e0212273. doi: 10.1371/journal.pone.0212273 (PMC6402636; doi:10.1371/journal.pone.0212273)
Supplement: S4 Table — File name: S4 Table. (DOCX) [file pone.0212273.s005.docx]

**S4 Table.** Demographic and clinical characteristics of study participants separated by smoking status^a^.

| **Characteristics** | **Smoking throughout pregnancy (n=43)** | **Non-smoking**  **(n=62)** | **p value** |
| --- | --- | --- | --- |
| **Maternal age at delivery (years)** | 30±6 (17- 41) | 32±5 (21- 41) | **0.03** |
| **Weeks of gestation at visit (weeks)** |  |  |  |
| A | 19±2 (14-23) | 19±2 (13-23) | 0.60 |
| B | 26±1 (24-30) | 26±1 (23-30) | 0.09 |
| C (n=42, 62) | 34±2 (30-40) | 34±1 (31-37) | 0.15 |
| Del (n=28, 27) | 38±2 (32-41) | 39±1 (35- 41) | 0.08 |
| **GA at delivery (weeks)** | 38±2 (32-42) | 39±2 (35-42) | **0.05** |
| **Preterm delivery (GA<37 weeks)** | 9 (21) | 8 (13) | 0.27 |
| **Infant SGA**^b^ **(n=42,61)** | 7 (17) | 8 (13) | 0.62 |
| **Race/Ethnicity** |  |  | **<0.001** |
| Indigenous/First Nations | 21 (49) | 5 (8) |  |
| Black/African Canadians | 0 (0) | 11 (17) |  |
| White/ Caucasian | 19 (44) | 30 (48) |  |
| Asian/Other | 3 (7) | 16 (25) |  |
| **Income <$15,000/year** | 28 (65) | 17 (27) | **<0.001** |
| **History of HCV infection**^c^ | 25 (81) | 2 (3) | **<0.001** |
| **Living with HIV** | 33 (77) | 31 (50) | **0.006** |
| **Substance use throughout pregnancy**^d^**(yes *vs.* no)** |  |  |  |
| Illicit drug^e^ | 13 (30) | 0 (0) | **<0.001** |
| Alcohol | 3 (7) | 5 (8) | 0.836 |

Data are presented as mean ± SD (range) or n (% of total) unless otherwise indicated. Abbreviations: GA, gestational age; SGA, Small for gestational age; Del; delivery; HCV, Hepatitis C Virus. ^a^ Substance use throughout pregnancy is defined as self-reported use of substance at ≥3 visits during pregnancy inclusive of the period prior to delivery.  ^b^ SGA infants have birth weights below the 10th percentile for infants of the same GA and sex. In this study, SGA is calculated according to the British Columbia (BC), Canada statistics provided by perinatal service BC. ^c^ History of HCV infection was defined as self-report of HCV+ status and/or a lab test result.^d^ Substance use throughout pregnancy is defined as self-reported use of substance at ≥3 visits during pregnancy inclusive of the period prior to delivery. ^e^ Illicit drug includes heroin, cocaine, opioids, amphetamines, benzodiazepenes and/or 3, 4-methylenedioxy-methamphetamine (MDMA).
