# Supplementary material for: Dynamics of leukocyte telomere length in pregnant women living with HIV, and HIV-negative pregnant women: A longitudinal observational study
Source: PLoS One. 2019 Mar 6;14(3):e0212273. doi: 10.1371/journal.pone.0212273 (PMC6402636; doi:10.1371/journal.pone.0212273)
Supplement: S5 Table — File name: S5 Table. (DOCX) [file pone.0212273.s006.docx]

**S5 Table.** Demographic, clinical, and laboratory characteristics of study participants separated by cohort.

| **Characteristics** | **All women**  **(n=105)** | |  | **WLWH**  **(n=64)** | |  | **HIV-negative women**  **(n=41)** | |  |
| --- | --- | --- | --- | --- | --- | --- | --- | --- | --- |
|  |  |  |  |  |  |  |  |  |  |
|  |  |  | **P** |  |  | **P** |  | | **P** |
|  | **CARMA**  **(n=33)** | **Pregnancy**  **(n=72)** | **value** | **CARMA**  **(n=23)** | **Pregnancy**  **(n=41)** | **value** | **CARMA**  **(n=10)** | **Pregnancy** | **value** |
|  |  |  |  |  |  |  |  | **(n=31)** |  |
|  |  |  |  |  |  |  |  |  |  |
| **Maternal age at delivery (years)** | 32± 5  (21-40) | 31 ± 6  (17-41) | 0.61 | 32 ± 5  (22-40) | 30 ± 6  (17-41) | 0.30 | 31 ± 5  (21-36) | 32 ± 5  (22-41) | 0.48 |
|  |  |  |  |  |  |  |  |  |  |
| **Preterm delivery**  **(GA<37 weeks)** | 4 (12) | 13 (18) | 0.44 | 4 (14) | 8 (20) | 0.83 | 0 (0) | 5 (16) | 0.17 |
|  |  |  |  |  |  |  |  |  |  |
| **Race/Ethnicity** |  |  |  |  |  |  |  |  |  |
| Indigenous/First Nations | 11 (3) | 15 (21) | 0.48 | 10 (43) | 13 (32) | 0.80 | 1 (10) | 2 (6) | 0.25 |
| Black/African Canadians | 4 (12) | 15 (21) |  | 4 (17) | 8 (20) |  | 0 (0) | 0 (0) |  |
| White/ Caucasian | 3 (19) | 8 (11) |  | 3 (13) | 8 (20) |  | 9 (90) | 22 (71) |  |
| Asian/Other | 15 (45) | 34 (47) |  | 6 (26) | 12 (29) |  | 0 (0) | 7 (23) |  |
| **Income <$15,000/year** | 15 (45) | 29 (40) | 0.61 | 12 (52) | 21 (51) | 0.94 | 3 (30) | 8 (26) | 0.79 |
| **History of HCV infection (yes *vs.* no) ^a^** | 12 (36) | 15 (21) | 0.09 | 12 (52) | 13 (32) | 0.11 | 0 (0) | 2 (6) | 0.41 |
| **Substance use throughout pregnancy ^b^(yes *vs.* no)** |  |  |  |  |  |  |  |  |  |
| Smoking ^b^ | 15 (45) | 28 (64) | 0.52 | 14 (61) | 19 (46) | 0.26 | 1 (10) | 9 (29) | 0.22 |
| Illicit drug ^c^ | 5 (15) | 8 (11) | 0.56 | 5 (22) | 4 (10) | 0.17 | 0 (0) | 4 (13) | 0.23 |
| Alcohol | 2 (6) | 6 (8) | 0.68 | 1 (4) | 2 (5) | 0.92 | 1 (10) | 4 (13) | 0.81 |
| **Duration of HIV infection at delivery (years)** | --- | --- | --- | 6.6 ± 5.2  (0.4-19.2) | 5.8 ± 4.1  (0.4-15.3) | 0.64 | --- | --- | --- |
| **Age at HIV diagnosis (years)** | --- | --- | --- | 25 ± 7  (3-36) | 24 ± 6  (2-35) | 0.45 | --- | --- | --- |
| **CD4+ nadir (cells/µl)** | --- | --- | --- | 285 ± 217  (10-800) | 292 ± 184  (20-910) | 0.64 | --- | --- | --- |
| **Detectable HIV pVL at delivery (>50 copies/mL) (n=29, 16)** | --- | --- | --- | 5 (38) | 3 (11) | 0.04 | --- | --- | --- |
| **cART exposure during pregnancy (weeks)** | --- | --- | --- | 25.9 ± 9.9  (8.7-40.1) | 24.6 ± 10.6  (0.3-41.7) | 0.85 | --- | --- | --- |
| **ART naïve pre-pregnancy** | --- | --- | --- | 9 (39) | 14 (34) | 0.69 | --- | --- | --- |
| **Conceived on cART** | --- | --- | --- | 6 (26) | 13 (23) | 0.63 | --- | --- | --- |
| **Received PI/r-based regimen during pregnancy** | --- | --- | --- | 21 (91) | 13 (32) | **<0.001** | --- | --- | --- |

Data are presented as mean ± SD (range) or n (% of total) unless otherwise indicated. Abbreviations: GA, gestational age; HCV, Hepatitis C Virus; CD4; cluster of differentiation 4; HIV pVL, HIV plasma viral load; cART, combination antiretroviral therapy. ^a^ History of HCV infection was defined as self-report of HCV+ status and/or a lab test result.^b^ Smoking throughout pregnancy is defined as self-reported use of tobacco and/or marijuana at ≥3 visits during pregnancy inclusive of the period prior to delivery. ^c^ Illicit drug includes heroin, cocaine, opioids, amphetamines, benzodiazepenes and/or 3, 4-methylenedioxy-methamphetamine (MDMA).
