# Supplementary material for: Dynamics of leukocyte telomere length in pregnant women living with HIV, and HIV-negative pregnant women: A longitudinal observational study
Source: PLoS One. 2019 Mar 6;14(3):e0212273. doi: 10.1371/journal.pone.0212273 (PMC6402636; doi:10.1371/journal.pone.0212273)
Supplement: S6 Table — Leukocyte telomere length (LTL) at visit A, B, and C separated by HIV status and at delivery and post-partum only for WLWH. (DOCX) [file pone.0212273.s007.docx]

**S6 Table.** Leukocyte telomere length (LTL) at visit A, B, and C separated by HIV status and at delivery and post-partum only for WLWH.

| **LTL** | **All women**  **(n=105)** | **WLWH**  **(n=64)** | **HIV-negative women (n=41)** | **P value** |
| --- | --- | --- | --- | --- |
| A (n=105, 64, 41) | 7.4 ± 0.9 (5.7-10.6) | 7.2 ± 0.8 (5.7-9.2) | 7.6 ± 0.9 (6.0-10.6) | **0.006** |
| B (n=105, 64, 41) | 7.5 ± 0.9 (5.0-10.0) | 7.4 ± 0.9 (5.0-10.0) | 7.6 ± 0.9 (6.1-10.0) | 0.28 |
| C (n=103, 62, 41) | 7.5 ± 0.9 (5.6-10.7) | 7.4 ± 0.9 (5.6-9.8) | 7.7 ± 0.9 (5.9-10.7) | 0.09 |
| Del (n=55) | 7.2 ± 1.0 (5.5-10.9) | 7.2 ± 1.0 (5.5-10.9) | --- | --- |
| P-P (n=59) | 7.2 ± 0.8 (5.6-9.6) | 7.2 ± 0.8 (5.6-9.6) | --- | --- |

Data are presented as mean ± SD (range). Abbreviations: WLWH, women living with HIV; LTL, leukocyte telomere length (LTL); Del; delivery; P-P, post-partum. TL comparisons between groups were done using either student t-test or Mann-Whitney test.
