# Supplementary material for: Dynamics of leukocyte telomere length in pregnant women living with HIV, and HIV-negative pregnant women: A longitudinal observational study
Source: PLoS One. 2019 Mar 6;14(3):e0212273. doi: 10.1371/journal.pone.0212273 (PMC6402636; doi:10.1371/journal.pone.0212273)
Supplement: S7 Table — File name: S7 Table. (DOCX) [file pone.0212273.s008.docx]

**S7 Table.** Multivariate analyses of the association between various factors and LTL in all participants and separated by HIV status.

| **Predictors** | **All women**  **(R^2^= 0.19) (n=105)** | | | **WLWH**  **(R^2^=0.23) (n=64)** | | | **HIV-negative women**  **(R^2^=0.40) (n=41)** | | |
| --- | --- | --- | --- | --- | --- | --- | --- | --- | --- |
|  | **β** | **95%CI** | **P Value** | **β** | **95%CI** | **P Value** | **β** | **95%CI** | **P Value** |
| **Maternal age at delivery (years)** | -0.01 | -0.10 – 0.08 | 0.86 | 0.05 | -0.05 – 0.16 | 0.29 | -0.09 | -0.25 – 0.07 | 0.27 |
| **Maternal age at delivery*Weeks of gestation** | -0.02 | -0.04 –  -0.00 | **0.01** | -0.04 | -0.07 –  -0.00 | **0.02** | -0.03 | -0.07 – 0.01 | 0.12 |
| **Weeks of gestation at Visit A** | --- | --- | **---** | --- | --- | **---** | -0.26 | -0.68 – 0.16 | 0.23 |
| **HIV status (yes *vs.* no)** | -0.35 | -0.87 – 0.17 | 0.19 | --- | --- | **---** |  |  |  |
| **History of HCV infection ^a^ (yes *vs.* no)** | 0.03 | -0.70 – 0.77 | 0.93 | --- | --- | **---** | **---** | **---** | **---** |
| **Smoking throughout pregnancy ^b^ (yes *vs.* no)** | -0.61 | -1.25 – -0.03 | 0.06 | -0.61 | -1.20 – -0.02 | **0.04** | **---** | **---** | **---** |
| **Received PI/r during pregnancy (yes *vs.* no)** | **---** | **---** | **---** | -0.64 | -1.25 – -0.05 | **0.03** | **---** | **---** | **---** |
| **Cohort (CARMA *vs.* Pregnancy)** | **---** | **---** | **---** | **---** | **---** | **---** | 0.57 | -0.36 – 1.49 | 0.23 |

Abbreviations: HCV, Hepatitis C Virus; PI, Protease inhibitor. ^a^ History of HCV infection was defined as self-report of HCV+ status and/or a lab test result.^b^ Smoking throughout pregnancy is defined as self-reported use of tobacco and/or marijuana at ≥3 visits during pregnancy inclusive of the period prior to delivery.
