# Supplementary figures and images for: Dynamics of leukocyte telomere length in pregnant women living with HIV, and HIV-negative pregnant women: A longitudinal observational study
Source: PLoS One. 2019 Mar 6;14(3):e0212273. doi: 10.1371/journal.pone.0212273 (PMC6402636; doi:10.1371/journal.pone.0212273)

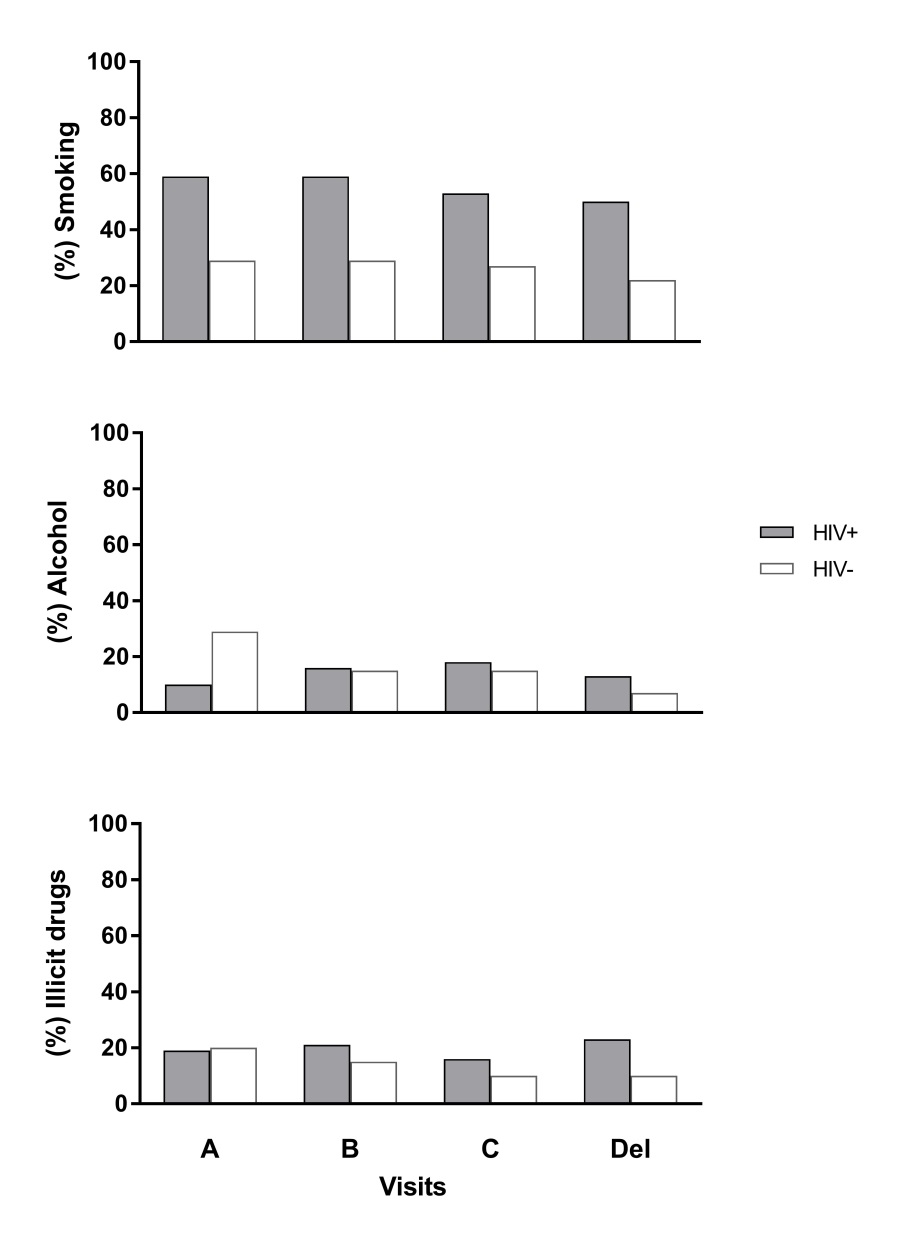


**S1 Fig.** Rate of substance use at each visit.

**HIV-negative**

**WLWH**

Supplement: S1 Fig — (DOCX) [file pone.0212273.s009.docx]
